# Associations between organised leisure-time activities and mental health problems in children

**DOI:** 10.1007/s00431-022-04591-9

**Published:** 2022-09-12

**Authors:** Mirte Boelens, Michel S. Smit, Dafna A. Windhorst, Harrie J. Jonkman, Clemens M. H. Hosman, Hein Raat, Wilma Jansen

**Affiliations:** 1 Department of Public Health, Erasmus MC, University Medical Center, PO BOX 2040, 3000 CA Rotterdam, The Netherlands; 2 Department of Cognitive Neuroscience, Donders Institute for Brain, Cognition and Behaviour, Radboud University Medical Center, Nijmegen, The Netherlands; 3 TNO Child Health, Leiden, The Netherlands; 4 Verwey-Jonker Institute, Utrecht, The Netherlands; 5 Department of Health Promotion, Maastricht University, Maastricht, The Netherlands; 6 Department of Clinical Psychology, Radboud University, Nijmegen, The Netherlands; 7 Hosman Prevention and Innovation Consultancy, Berg en Dal, The Netherlands; 8 Department of Social Development, Municipality of Rotterdam, PO BOX 70032, 3000 LP Rotterdam, The Netherlands

**Keywords:** Psychosocial, Sports, Extracurricular, Structured activities, Well-being, Leisure

## Abstract

Previous studies conducted mainly among adolescents have found associations between participation in sport organised leisure-time activities (OLTAs) and mental health problems (MHP). Fewer research studies have been performed to primary school-aged children and to organised non-sport OLTAs. Therefore, the objective is to examine whether there is an association between participation in sport and non-sport OLTAs and a high risk of MHP in 4- to 12-year-olds. Data were used on 5010 children from a cross-sectional population-based survey conducted between May and July 2018 in Rotterdam, the Netherlands. Associations between sport OLTAs, non-sport OLTAs and breadth of OLTAs and a high risk of MHP were explored using logistic regression models adjusting for sociodemographic characteristics, stressful life events and physical activity. Of all children, 58% participated in sport OLTAs and 22% in non-sport OLTAs. The proportion of children with high risk of MHP among participants in sport OLTAs is smaller than among non-participants (OR 0.66, 95% CI: 0.53, 0.81). The proportion of children with high risk of MHP among participants in non-sport OLTAs is smaller than among non-participants (OR 0.69, 95% CI: 0.53, 0.91). The proportion of children with a high risk of MHP among participants in 1 category of OLTAs (OR 0.61, 95% CI: 0.49, 0.76) and in 2–5 categories of OLTAs (OR 0.48, 95% CI: 0.32, 0.71) is smaller than among non-participants.

*  Conclusion*: The proportion of children with high risk of MHP among participants in OLTAs is smaller than among non-participants.

**Table:** 

**What is Known:** *• Around 10–-20% of children and adolescents experiences mental health problems*. *• Sport organised leisure-time activities have been found to be associated with a lower risk of mental health problems in adolescents*.	
**What is New:** *• The proportion of children with a high risk of mental health problems in participants in organised leisure-time activities is smaller than among non-participants*. *• The proportion of children with a high risk of mental health problems in participants with a higher breadth of organised leisure-time activities is smaller compared to non-participants*.

**Supplementary Information:**

The online version contains supplementary material available at 10.1007/s00431-022-04591-9.

## Introduction

Around 10–20% of children and adolescents experience mental health problems (MHP) [1]. First onset usually occurs during childhood or adolescence [2]. MHP in children include but are not limited to anxiety disorders, depressive disorders, attention-deficit hyperactivity disorder (ADHD) or disruptive behaviour disorders [3]. Early intervention can reduce or prevent mental health problems in later life [1]. Hence, gaining more insight in possible modifiable factors contributing to good mental health in childhood is important.

Participating in organised leisure-time activities (OLTAs) may contribute to good mental health in childhood [4]. OLTAs are characterised by having a certain structure, schedule, tend to have clearly defined goals and rules and are focused on skill building [5, 6]. Examples of categories are sport, scouting and theatre lessons. Features that could be present in OLTAs that previously have been found to improve mental health are as follows: safe and appropriate peer interactions, structure and adult supervision, forming of supportive relationships with peers and emphasis on inclusion and a sense of belonging, emphasis on positive social norms, support of efficacy and mattering and skill-building [7]. Moreover, OLTAs can include physical activity as possible feature that could improve child mental health, such as sport OLTAs and scouting. Other kinds of OLTAs may include physical activity (e.g. scouting) but not necessarily or in the same amount. The positive youth development theory (PYD), grounded in the socio-ecological theory, postulates that OLTAs offer opportunities for children to develop relationships and to engage in activities that increase their competence, confidence, connection, character and caring. This could lead to better emotion regulation, positive connections with peers, adults and the larger community and consequently a better mental health [8–13]. The breadth, intensity, type, duration and engagement of children in OLTAs might also play a role [5].

Studies reported associations of participation in team and other types of sport OLTAs with better youth mental health [14, 15]. Non-sport OLTAs could also contribute to good child mental health [4–6]. Positive contributions to child mental health have been found but most earlier studies focused on adolescents [16–18]. Research to associations of participating in OLTAs with mental health in primary school-aged children is limited [14, 19–24] Associations of participation in OLTAs were mixed. Some of these studies found no association [21, 23]. However, other studies performed in children reported an association between participation in OLTAs and better mental health [14, 19, 20, 22, 24]. Prevention in this particular age group possibly contributes to reducing the risk of MHP among adolescents. Therefore, we aim to examine associations between participating in sport OLTAs, non-sport OLTAs and of the breadth of OLTAs and MHP in a population-based sample of 4- to 12-year-olds. We hypothesised that the proportion of children with a high a risk on MHP among participants in OLTAs is smaller than among non-participants. We studied sport and non-sport OLTAs separately because we hypothesised that the association of sport OLTAs might be different from the association non-sport OLTAs with mental health as was found in previous research in adolescents [16].

## Methods

### Study design

This cross-sectional study is performed using anonymous data from a Dutch Public Health survey carried out in 2018 by the municipal public health service in Rotterdam, the Netherlands. This survey was conducted in the context of performing statutory tasks (Public Health Act Netherlands). Observational research with anonymous data does not fall within the ambit of the Dutch Act on research involving human subjects and requires no approval of an ethics review board. The Dutch Code of Conduct for Medical Research allows using anonymous survey data for research purposes without an explicit informed consent [25]. The Dutch Public Health survey that was administered consists of questions about sociodemographic information, mental health, diet, physical health, physical activity, OLTAs, social support, care-use and stressful life events. Random probability sampling from the municipal population register stratified by neighbourhood was performed to create a sample of parents of 0- to 12-year-olds for the Dutch Public Health survey. Children living in healthcare institutions were excluded. All parents were living in Rotterdam when the survey was administered. Parents/caregivers received invitations for one child only (i.e. only one child from children living on the same address was included. The first child that was selected entered the sample.) No further selection criteria were used. Hardcopy invitation letters included information about privacy, content, aim, anonymity and login details for the online questionnaire. A toll-free telephone number was provided for additional questions. Hardcopy questionnaires were enclosed with the first reminder and could be requested in Dutch, English or Turkish. The main caregiver filled out the questionnaire. Non-responders were contacted by telephone and were offered extra help in completing the questionnaire. Parents/caregivers were free to refuse participation by not filling out the questionnaire. In total, 7702 parents/caregivers of 0- to 12-year-olds responded to the Dutch Public Health survey. The response rate was 34% and did not differ upon age or gender of the children.

### Study population

**Fig. 1:**
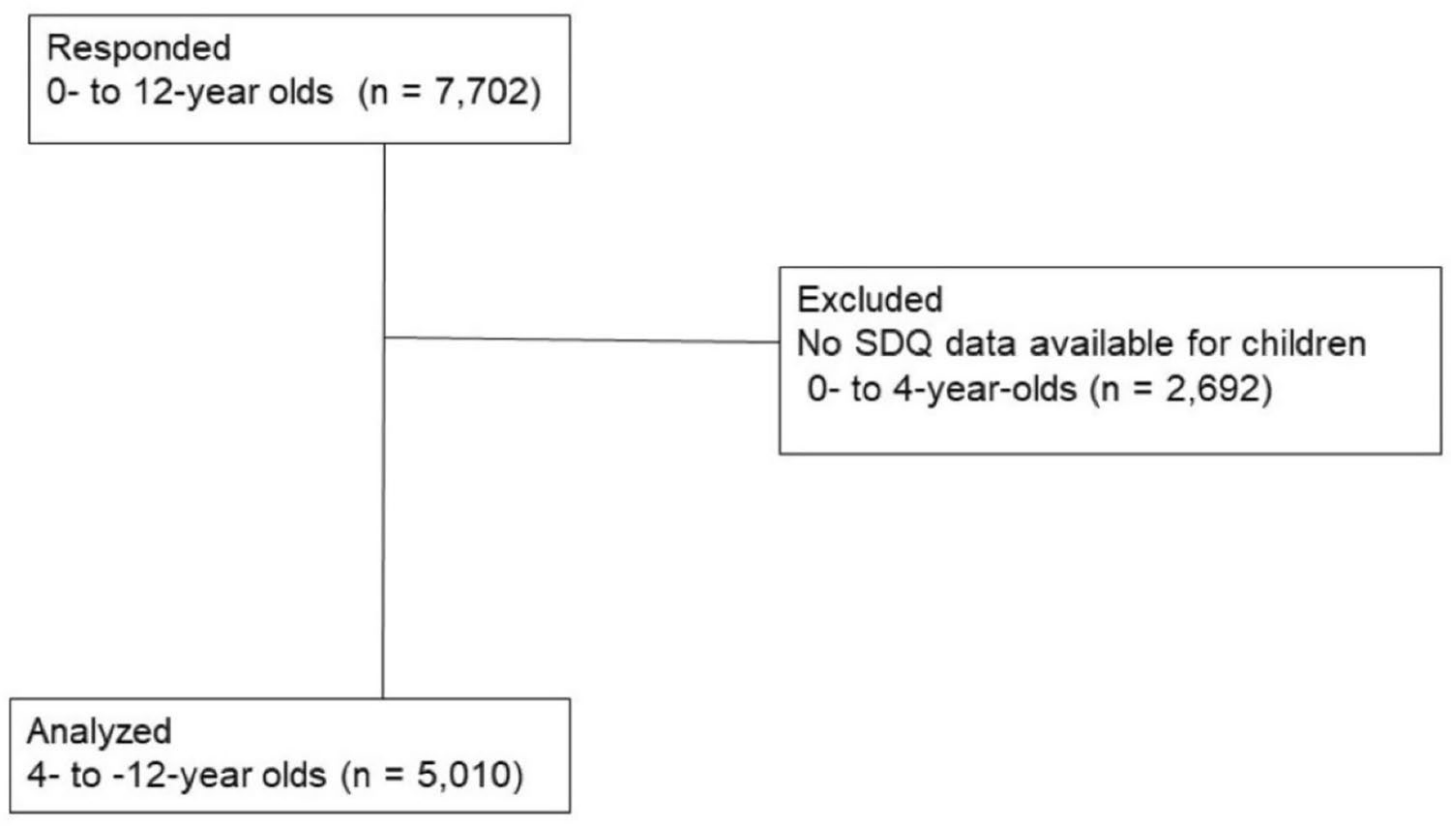
Flow diagram of study population

For our study, we used data about 4- to 12-year-olds (*n* = 5010) as the strengths and difficulties questionnaire (SDQ) to measure risk of MHP was not assessed in younger children. See Fig. 1 for an overview.

### Measurements

#### Explanatory variables

OLTA participation was measured by the question: ‘*Which associations or organizations is your child a member of?*’ Parents could choose between categories of OLTAs. The response option was binary (i.e. parents could either answer ‘Yes’ or ‘No’ for each of the OLTAs). Multiple answers were possible:

1. Sport associations;
2. Singing, music or theatre clubs/lessons;
3. Scouting;
4. Craft club;
5. Different kind of organisation;
6. None.

We computed three variables out of these items: sport OLTAs, non-sport OLTAs and breadth of OLTAs. Participation in sport OLTAs was based on item 1 and was categorised as ‘*Sport OLTA participation*’ and ‘*No sport OLTA participation*’ using the latter as reference group. Participation in non-sport OLTAs was based on items 2, 3, 4 and 5 and categorised as ‘*non-sport OLTA participation*’ and ‘*No non-sport OLTA participation*’ using the latter as reference group. We computed different variables for sport and non-sport OLTAs because as they may differ with respect to physical activity levels and also for comparison purposes with previous studies [26]. We chose to combine all non-sport OLTAs to ensure sufficient children in each category. In some of the non-sport OLTAs, only a small amount of children were participating (i.e. singing, music, theatre = 11.2%, scouting = 2.0%, craft club = 2.5%, other = 8.5%). The breadth of OLTAs was based on all six items and categorised as ‘*2–5 categories*’, ‘*1 category*’ and ‘*None*’, using the latter as reference group. This variable includes different categories of OLTAs to capture the breadth. We chose for this categorisation to ascertain sufficient children in each category (i.e. 1 category = 55.1%, 2 categories = 11.0%, 3 categories = 1.3%, 4 categories = 0.1% and 5 categories = 0.02%).

#### Study outcome

We computed the risk of MHP using the parent-reported strengths and difficulties questionnaire (SDQ) which was embedded in the public health survey. The SDQ measures risk of MHP but not MHP. The SDQ is a validated questionnaire (the SDQ has good validity compared to the Child Behaviour Checklist in the original and in Dutch versions as well as good overall reliability) to measure risk of MHP and consists of five domains: emotional problems, conduct problems, hyperactivity, peer problems and prosocial behaviour [27–30]. The total difficulties score was calculated by adding the scores of all domains except for prosocial behaviour (range = 0–40) (Cronbach’s *α* = 0.73). We dichotomised the total difficulties score using age-dependent cut-off scores to either ‘*High risk of MHP*’ or a ‘*Normal score*’ with the latter as reference group. For 4- to 7-year-olds, a total difficulties score of ≥ 15 indicates risk of MHP and for 7- to 12-year-olds a cut-off is ≥ 14 indicates risk of MHP [27, 28].

#### Covariates

Gender, age, family situation, parental education, migrant status, perceived financial difficulties, current stressful life events and adequate physical activity were selected based on theory and previous research and were derived from the survey [4–6]. Gender or age can affect participation rates and have found to be associated with MHP [4, 5, 26]. Minority- and disadvantaged groups generally participate less in OLTAs [5, 31]. Moreover, they may be more at risk to develop MHP just as children who experience stressful life events, who live in low SES families or not in a two-parent family [32–34]. Physical activity is a key aspect of sport OLTAs and could be present in non-sport OLTAs and may be associated with better mental health in children [35]. Age was measured continuously in years. Gender was measured as ‘*Girl*’ or ‘*Boy*’. Family status was measured as ‘*Two-parent family*’ or ‘*Single-parent/other type of family*’. Parental educational level was defined as highest parental educational level obtained and categorised as ‘*lower education*’ (no education, primary school or ≤ 4 years general secondary school), ‘*intermediate education*’ (> 4 years general secondary school or intermediate vocational training) and ‘*higher education*’ (higher vocational training, university degree or higher) [36]. Parent-reported migrant status of the child was measured as ‘*Non-Western*’ or ‘*Non-Dutch Western*’ and ‘*Dutch*’. A non-Western migrant status was assigned when the child itself or either one or both of the parents were born in a non-Western country. A non-Dutch Western migrant status was assigned when the child itself or either one or both of the parents were born in a Western country different from the Netherlands [37]. Perceived financial difficulties was measured by the question: ‘Did you experience any difficulties in making ends meet in the past twelve months with your household income?’ Perceived financial difficulties had four answer categories and was dichotomised as ‘*No financial difficulties*’ (‘*No*’ and ‘*No but I have to think about my expenses*’) and ‘*Financial difficulties*’ (‘*Yes a little*’ and ‘*Yes*’). Current stressful life events was measured by eighteen stressful life events (e.g. ‘*Divorce of parents*’). In case of ≥ 1 stressful life events currently experienced, this variable was categorised as ‘*Yes*’. In case of no current stressful life events, this variable was categorised as ‘*No*’. Physical activity was measured by eight questions about five physical activity domains: commuting to school, by outdoor-play, by physical education or swimming lessons at school and by sport club membership. All questions concerned the past week. Parents answered the number of days for an activity (answer categories ‘*1 day*’, ‘*2 days*’, ‘*3 days*’, ‘*4 days*’, ‘*5 days*’, ‘*6 days*’, ‘7 days’, ‘*none*’, ‘*not the past week but normally yes*’) followed by a question on the minutes per day (answer categories ‘ < *10 min*’, ‘*10–20 min*’, ‘*20–30 min*’, ‘*30–60 min*’, ‘ > *60 min*’ or ‘*Not applicable*’). These physical activity questions are used by health services in the Netherlands and scored in minutes per week, which was changed to hours per day for the present study.

### Statistical analyses

Descriptive statistics were calculated and chi-square and Mann–Whitney *U* tests were performed to test for differences between OLTA participation. Multiple imputation (*m* = 10) with the fully conditional specification method was used for missing values of variables (total 0.7% ranging from 0.3 to 4.0%) using data on explanatory variables, outcome variables and covariates as predictors [38]. Multivariable logistic regression analyses were performed to examine associations between participating in OLTAs and a high risk of MHP. We computed three models separate for sport OLTAs and for non-sport OLTAs and consecutively a fourth model which is a combined model for both explanatory variables (sport and non-sport OLTAs). Model 1 was a crude model. Model 2 was adjusted for age, gender, parental education, family status, perceived financial difficulties and migrant status of the child. Model 3 was additionally adjusted for current stressful life events and physical activity. Model 4 is model 3 and additionally mutually adjusted for sport OLTAs and non-sport OLTAs to examine independent associations (combined model). Multivariable logistic regression analyses to examine the association between the breadth of OLTAs and a high risk of MHP were also performed. Three models similar to models 1, 2 and 3 for sport and non-sport OLTAs were computed. To examine whether the impact differed upon groups with different characteristics, interactions between age, gender, family status, migrant status, perceived financial difficulties and sport OLTAs, non-sport OLTAs and breadth of OLTAs were tested by adding the product terms of the explanatory variables with each of the potential effect modifiers separately to the full model (model 4 for sport and non-sport OLTAs and model 3 for breadth of OLTAs) [5, 13, 16]. Interactions between participating in sport and non-sport OLTAs were tested likewise [5, 13]. Interactions were considered present at a significance level of *p* < 0.05 and none was found (Table S1).

#### Sensitivity analyses

Sensitivity analyses using a complete-case dataset were conducted for comparison. Missing-value analysis was performed using descriptive characteristics and chi-square or Mann–Whitney *U* tests for differences between children without and with missing values. These analyses are included in the supplementary material (Tables S2 and S3 are about complete-case analyses, and Table S4 is about missing-value analysis).

Two-tailed analyses were performed using IBM SPSS statistics version 25 (SPSS Inc., Armonk, NY, USA) and *p* values < 0.05 were considered statistically significant.

## Results

Table 1 shows the population characteristics. Median age was 8.0 (IQR = 6.0–10.0). The sample consisted of 48% girls. Of all children, 58% participated in sport OLTAs, 22% participated in non-sport OLTAs and 32% in none. In our sample, 55% participated in 1 category of OLTAs and 13% in 2–5 categories of OLTAs. Generally, more boys participated in organised sport OLTAs and more girls in non-sport OLTAs. Children with lower educated parents, with parents perceiving financial difficulties and with a non-Western migrant status participated less in sport OLTAs.

**Table 1:** Characteristics of the study sample

	**Total population** ***N* = 5010**	**Organised leisure-time activities (OLTAs)**					
		**No sport OLTAs** ***N* = 2117 (42.0%)**^**a**^	**Sport OLTAs** ***N* = 2865 (58%)**^**a**^	***p* value**	**No non-sport OLTAs** ***N* = 3886 (78%)**^**a**^	**Non-sport OLTAs** ***N* = 1096 (22%)**^**a**^	***p* value**
**Breadth of organised leisure-time activities (OLTAs)**^**a**^				** < 0.001**			** < 0.001**
In 2–5 categories	621 (13%)	41 (1.9%)	580 (20%)		0 (0%)	621 (57%)	
In 1 category	2760 (55%)	475 (22%)	2285 (80%)		2285 (59%)	475 (43%)	
None	1601 (32%)	1601 (76%)	0 (0%)		1601 (41%)	0 (0%)	
**Age, median (IQR)**	8.0 (6.0–10.0)	7.0 (5.0–9.0)	8.0 (6.0–10.0)	** < 0.001**	7.0 (5.0–9.0)	8.0 (6.0–10.0)	** < 0.001**
**Gender**				** < 0.001**			** < 0.001**
Boy	2571 (52%)	998 (47%)	1573 (55%)		2153 (55%)	418 (38%)	
Girl	2411 (48%)	1119 (53%)	1292 (45%)		1733 (45%)	678 (62%)	
**Parental education**^b^				** < 0.001**			** < 0.001**
Higher	2452 (51%)	789 (39%)	1663 (60%)		1802 (48%)	650 (62%)	
Intermediate	1543 (32%)	752 (37%)	791 (28%)		1259 (34%)	284 (27%)	
Lower	790 (17%)	468 (23%)	322 (12%)		668 (18%)	122 (12%)	
**Financial difficulties**^c^				** < 0.001**			0.45
No	4157 (85%)	1667 (80%)	2508 (88%)		3253 (85%)	922 (85%)	
Yes	753 (15%)	420 (20%)	333 (12%)		596 (15%)	157 (15%)	
**Migrant status**^d^				** < 0.001**			** < 0.001**
Dutch	2277 (45%)	714 (34%)	1563 (55%)		1731 (45%)	546 (50%)	
Western migrant	650 (13%)	299 (14%)	351 (12%)		478 (12%)	172 (16%)	
Non-Western migrant	2029 (41%)	1087 (52%)	942 (33.0%)		1658 (43%)	371 (34%)	
**Family status**^e^				** < 0.001**			1.0
Two-parent	3700 (75%)	1499 (72%)	2201 (77%)		2889 (75%)	811 (75%)	
Other	1245 (25%)	595 (28%)	650 (23%)		972 (25%)	273 (25%)	
**Current stressful life events**^f^				** < 0.001**			0.29
No	3745 (75%)	1535 (73%)	2210 (77%)		2935 (76%)	810 (74%)	
Yes	1222 (25%)	573 (27%)	647 (23%)		940 (24%)	282 (26%)	
**Physical activity, median (IQR)**^g^	1.7 (1.1–2.4)	1.4 (0.9–2.1)	1.8 (1.3–2.5)	** < 0.001**	1.7 (1.1–2.4)	1.6 (1.1–2.3)	0.46
**Mental health problems (MHP)**^**h**^				** < 0.001**			**0.002**
No	4487 (91%)	1853 (88%)	2634 (92%)		3477 (90%)	1010 (93%)	
Yes	470 (9.5%)	253 (12%)	217 (7.6%)		394 (10%)	76 (7.0%)	

Chi-square tests were used to test for differences in categorical variables and Mann–Whitney *U* tests for continuous variables. Percentages are represented by column percentages. Valid percentages are reported. **Bold** indicates significance (*p* value < 0.05). For this study, we used data from a survey conducted between May and July in Rotterdam, the Netherlands, from 5010 children aged 4–12-years-old

^a^28 (0.6%) missing

^b^201 missing (4.0%)

^c^71 missing (1.1%)

^d^26 missing (0.5%)

^e^37 missing (0.7%)

^f^17 missing (0.4%)

^g^29 missing (0.6%)

^h^28 missing (0.6%)

Table 2 presents associations between OLTAs and a high risk of MHP. After adjustment for confounders, the proportion of children with a high risk of MHP among participants in sport OLTAs (OR 0.65, 95% CI: 0.53, 0.81) is smaller compared to non-participants (model 4). The proportion of children with a high risk of MHP among participants in non-sport OLTAs (OR 0.69, 95% CI: 0.53, 0.91) is smaller than among non-participants (model 4).

**Table 2:** Associations of organised leisure-time activities (OLTAs) and risk of mental health problems (MHP) in 5010 children

	Model 1 OR (95% CI)	Model 2 OR (95% CI)	Model 3 OR (95% CI)	Model 4 OR (95% CI)
**Sport organised leisure-time activities**				
Yes	**0.61 (0.50, 0.73)**	**0.61 (0.50, 0.76)**	**0.67 (0.54, 0.83)**	**0.65 (0.53, 0.81)**
No	Ref	Ref	Ref	Ref
* Nagelkerke R-square*	0.011	0.073	0.095	0.098
**Non-sport organised leisure-time activities**				
Yes	**0.67 (0.52, 0.88)**	**0.75 (0.58, 0.98)**	**0.73 (0.56, 0.96)**	**0.69 (0.53, 0.91)**
No	Ref	Ref		Ref
* Nagelkerke R-square*	0.004	0.066	0.091	0.098

**Bold** indicates significance (*p* value < 0.05). Model 1 is a crude unadjusted model. Model 2 is adjusted for sociodemographic variables (i.e. age, gender (ref = boy), parental education (ref = higher), perceived financial difficulties (ref = no), family status (ref = two-parent), migrant status (ref = Dutch)). Model 3 is model 2 and additionally adjusted for physical activity and stressful life events (ref = no). Model 4 is model 3 and additionally (mutually) adjusted for organised sport or non-sport activities (i.e. independent association). Nagelkerke *R*-square is reported based on imputation 10. For this study, we used data from a survey conducted between May and July in Rotterdam, the Netherlands, from 5010 children aged 4–12-years-old

Table 3 presents associations between the breadth of OLTAs and a high risk of MHP. The proportion of children with a high risk of MHP among participants participating in 1 (OR 0.61, 95% CI: 0.49, 0.76) or in 2–5 (OR 0.48, 95% CI: 0.32, 0.71) categories is smaller than among non-participants (model 3).

**Table 3:** Associations of number of the breadth of organised leisure-time activities (OLTAs) and mental health problems (MHP) in 5010 children

	Model 1 OR (95% CI)	Model 2 OR (95% CI)	Model 3 OR (95% CI)
**Breadth of organised leisure-time activities (OLTAs)**			
In 2–5 categories	**0.40 (0.28, 0.58)**	**0.46 (0.31, 0.68)**	**0.48 (0.32, 0.71)**
In 1 category	**0.57 (0.47, 0.70)**	**0.56 (0.45, 0.69)**	**0.61 (0.49, 0.76)**
None	Ref	Ref	Ref
* Nagelkerke R-square*	0.018	0.079	0.101

**Bold** indicates significance (*p* value < 0.05). Model 1 is a crude unadjusted model. Model 2 is adjusted for sociodemographic variables (i.e. age, gender (ref = boy), parental education (ref = higher), perceived financial difficulties (ref = no), family situation (ref = two-parent), migrant status (ref = Dutch)). Model 3 is model 2 and additionally adjusted for physical activity and stressful life events (ref = no). Nagelkerke *R*-square is reported based on imputation 10. For this study, we used data from a survey conducted between May and July in Rotterdam, the Netherlands, from 5010 children aged 4–12-years-old

Complete-case analyses yielded similar estimates (Tables S2 and S3). Participating in non-sport OLTAs was non-significant in model 2 and model 3.

Comparisons between children with complete data (*n* = 4716) and with missing values (*n* = 294) on included variables indicated differences for family situation, parental education, perceived financial difficulties, migrant status, risk of MHP, age, sport OLTAs and breadth of OLTAs (Table S4).

## Discussion

The aim of this study was to examine associations between participating in OLTAs and risk of MHP in a population-based sample of 4- to 12-year-olds. We demonstrated that the proportion of 4- to 12-year-olds with a high risk of MHP among participants in sport or non-sport OLTAs is smaller compared to non-participants. The proportion of children with a high risk of MHP among participants in 1 category of OLTAs is smaller compared to non-participants. The proportion of children with a high risk of MHP among participants in 2–5 categories (i.e. higher breadth) of OLTAs was even smaller compared to non-participants.

The findings of this study support the hypothesis postulated by the PYD-theory that participation in OLTAs could improve academic, psychological, social and behavioural outcomes in youth and should therefore be encouraged [5]. This is because according to the PYD-theory OLTAs may offer a opportunities for children to develop relationships and to engage in activities that increase their competence, confidence, connection, character (e.g. respecting societal and cultural rules, sense of morality and integrity) and caring (i.e. the so-called 5 Cs) leading to better emotion regulation and positive connections with peers, adults and others [8–13]. This in turn could be protective for their mental health. Our findings are in agreement with earlier studies that also found an association between OLTAs and mental health in children and adolescents [14, 19, 20, 22, 24, 39]. In our study sample, 32% was not participating in any OLTA. Of all children, 58% participated in sport OLTAs and 22% in non-sport OLTAs. This is consistent with a study among Canadian 6- to 12-year-olds in which 39% was not participating in OLTAs [18]. The HBSC studies among nine nationally representative samples of 11-, 13- and 15-year-olds showed that 18% of the children was not participating in OLTAs but these children were somewhat older [26].

Sport OLTAs include several features mentioned by the PYD-theory that have been found to improve mental health. Due to these features, OLTAs could also contribute to good mental health. For example by offering children a place to develop executive functioning which is a precursor of behavioural regulation in childhood [40, 41]. Neville et al. also postulated that the structured, scheduled meetings, role-based, rule-governed and goal-oriented nature of these activities make them suitable for promoting beneficial developmental outcomes, such as behavioural regulation [22]. In their study, they observed that for boys with development delays, regular participation in sport OLTAs was longitudinally associated with a relative decrease in behavioural difficulties [22]. Indeed, previous studies into sport OLTAs already reported associations with better mental health in children. However, most did not adjust for physical activity [16, 42, 43]. It has been observed that physical activity itself is associated with child mental health [35]. Physical activity is also a one of the foundation stones of most of the sport OLTAs. A consequence is that it is impossible to differentiate between benefits due to participating in sport OLTAs or due to physical activity in these studies. We have adjusted for physical activity in our analyses and still found that the proportion of children with high risk of MHP among participants in sport OLTAs is smaller than among non-participants. The finding that participating in sport OLTAs is negatively associated with risk of MHP among participants, besides by increasing physical activity, supports the PYD-theory [8–12, 44]. Also in another study, lower total difficulties scores on the SDQ and less internalising problems were observed among children participating in sport OLTAs compared to non-participants after adjustment for physical activity [14].

Similarly, non-sport OLTAs also consist of several features that could contribute to good mental health and share features similar to features of sport OLTAs. Therefore, also participation in non-sport OLTAs could contribute to good mental health. Indeed, it has been found in previous research that participation in non-sport OLTAs is associated with conduct problems mediated by social skills [24]. In adolescents, it has been found to be associated with lower emotional-anxiety, higher pro-social behaviour and higher self-image just as participation in sport OLTAs [45]. That non-sport OLTAs are associated with mental health in children is supported by the findings in our study because the proportion of children with high risk of MHP among participants in non-sport OLTAs is smaller than among non-participants. This is in agreement with some previous studies that observed that participation in non-sport OLTAs might be associated with better mental health in children but research is inconclusive [18, 26, 46]. Among 27,121 Canadian 6- to 12-years-olds, no differences in mental health were found between children who did or did not participate in educational programs, arts/music and individual sport [18]. A possible explanation may be that they included children participating in educational programs to meet parental expectations leading to lower levels of mental health [18]. Contrary, data from the HBSC studies observed that both sport and non-sport OLTAs were associated with better mental well-being and fewer psychological complaints [26].

Previously, concerns were raised that participation in more OLTAs does not contribute to better mental health but in fact could increase risk of MHP [47]. A higher breadth of OLTAs was hypothesised as too time-consuming, an indication of parental pressure, costing to much free time and being too competitive and thus leads to poor developmental outcomes. This is also known as the over-scheduling hypothesis [47]. In our sample, the proportion of children with a high risk of MHP among participants in 2–5 categories of OLTAs was smaller than among participants in 1 category and among non-participants. However, confidence intervals of the estimates were overlapping. Most of the children participating in 2–5 categories of OLTAs participated in 2 categories; thus, we could not study whether there was some form of over-scheduling. Other studies reported associations of a higher breadth of OLTAs with better mental health [47, 48]. This could be because it leads to more developmental opportunities in different contexts [47, 48].

Strengths of this study are the population-based setting, large sample size and validated questionnaire for assessment of the risk of MHP. We adjusted for physical activity, showing that the associations of participating in sport OLTAs with mental health possibly also originates from another pathway than through physical activity itself. This study also has some limitations. Due to the cross-sectional design, no causation or temporal direction can be established. We adjusted for several covariates but residual confounding might be present because of incompletely or unmeasured confounders such as socioeconomic status indicators. Fewer disadvantaged children participated in OLTAs and we could only adjust for parental education, perceived financial difficulties and migrant status. The survey data were not nationally representative possibly reducing the generalisability. The response rate was 34%, which makes the study prone to selection bias, but low response rates do not automatically introduce bias in estimates or limit generalisability [49]. Moreover, surveys in the same Dutch city have similar response rates [50]. Data were collected in 2018 before the COVID-19 pandemic. Therefore, the data and the results may not reflect the situation after the COVID-19 outbreak. Generalising these results to a situation after the COVID-19 outbreak should be done with caution. Data about MHP were parent-reported. This could lead to social-desirable answers from parents about their children. Furthermore, the mental health state of the parents could also influence the answers on the SDQ. A study from 2001 observed that mentally healthy mothers might underreport and that parents with MHP might over report MHP in children [51]. We have no information about the frequency, intensity or duration or if OLTAs were individual or group-based. Finally, the Nagelkerke *R*^2^ values of our models were relatively low, even in the adjusted models. The Nagelkerke *R*^2^ is a relative measure that indicates how well the model explains the data. Even though we observed significant associations, we should interpret our models with caution.

### Public health implications

The findings of this study demonstrate that the proportion of children with a high risk of HP among participants in both sport and non-sport OLTAs is smaller compared to non-participants. This supports earlier studies that observed associations between participation in OLTAs and mental health in adolescents and the few studies that examined this in children. As we adjusted for physical activity, our study indicates that the beneficial effects of OLTAs might originate from other features specific for OLTAs.

Preventive policies could contribute to good mental health by stimulating more children to participate in OLTAs. Municipalities can increase the availability and amount of local clubs/associations and schools could offer additional extracurricular OLTAs [52].

### Future research

We recommend studying OLTAs more in depth by focusing on specific activities and its features [53, 54]. For example whether the association with mental health in children differs upon the content of the design (how inclusive it is, degree of structure), content (to which degree does it integrates cognitive, socio-emotional, motor skills), the environment (e.g. extracurricular at school or community) or on the resources (indoor/outdoor, level of adult involvement, equipment) of the activity [54]. Furthermore, we recommend studying aspects of OLTAs such as characteristics of trainers and group of peers participating in OLTAs [12]. Studying associations between OLTAs and mental health in children with developmental and/or physical disabilities is also recommended as these children are generally excluded from research [54]. It is of particular importance that future studies also examine potential determinants for participation/non-participation and how policymakers can ensure that more children can and will participate in such activities.

## Conclusions

In this population-based sample of 5010 primary school-aged children, the proportion of children with a high risk of MHP among participants in OLTAs is smaller compared to non-participants. This was observed for participants in sport OLTAs as well as for participants in non-sport OLTAs irrespective of physical activity levels. In this sample of 4- to 12-year-olds, an association of a higher breadth of OLTAs with a lower risk of MHP in 4- to 12-year-old children was also observed.

## Supplementary Information

Below is the link to the electronic supplementary material.

Supplementary file1 (DOCX 24 KB)

## Data Availability

The data used in this study are protected by the Municipal Health Service of Rotterdam. Data are available under request via gezondheidsmonitorbco@rotterdam.nl.

## Abbreviations

MHP: Mental health problems
PYD: Positive youth development
SDQ: Strengths and difficulties questionnaire
