# Supplementary material for: Associations between organised leisure-time activities and mental health problems in children
Source: Eur J Pediatr. 2022 Sep 12;181(11):3867–77. doi: 10.1007/s00431-022-04591-9 (PMC9467418; doi:10.1007/s00431-022-04591-9)
Supplement: Supplementary file 1 — Supplementary file1 (DOCX 24 KB) [file 431_2022_4591_MOESM1_ESM.docx]

| **Table S1.** Tests for interaction effects | |
| --- | --- |
|  | p-value logistic regression |
| **Sport and non-sport organized leisure-time activities** |  |
| Sport organized leisure-time activities*non-sport organized leisure-time activities | 0.17 |
| **Sport organized leisure-time activities** |  |
| Sport organized leisure time activities *gender | 0.39 |
| Sport | 0.39 |
| Sport organized leisure-time activities* family status | 0.60 |
| Sport organized leisure-time activities * perceived financial difficulties | 0.93 |
| Sport organized leisure-time activities * migrant status | 0.46 |
| **Non-sport organized leisure-time activities** |  |
| Non-sport organized leisure-time activities* gender | 0.72 |
| Non-sport organized leisure-time activities * age | 0.95 |
| Non-sport organized leisure-time activities * family status | 0.18 |
| Non-sport organized leisure-time activities * perceived financial difficulties | 0.25 |
| Non-sport organized leisure-time activities* migrant status | 0.52 |
| **Number of categories of organized leisure-time activities** |  |
| Number of categories of organized leisure-time activities *gender | 0.50 |
| Number of categories of organized leisure-time activities * age | 0.35 |
| Number of categories of organized leisure-time activities* family status | 0.65 |
| Number of categories of organized leisure-time activities * perceived financial difficulties | 0.34 |
| Number of categories of organized leisure-time activities * migrant status | 0.42 |
| Tests for interaction were performed in model 4 (adjusted for age, gender (ref=boy), parental education (ref=higher), perceived financial difficulties (ref=no), family status (ref=two-parent), migrant status (ref=Dutch), physical activity, and stressful life events (ref=no) and additionally (mutually) adjusted for organized sport or non-sport activities (i.e. independent association)) for sport and organized non-sport organized leisure-time activities and in model 3 (adjusted for age, gender (ref=boy), parental education (ref=higher), perceived financial difficulties (ref=no), family status (ref=two-parent), migrant status (ref=Dutch), physical activity, and stressful life events (ref=no)) for number of categories of organized leisure-time activities. For this study we used data from a survey conducted between May-July in Rotterdam, the Netherlands from 5,010 children aged 4-12-years old. | |

| **Table S2.** Complete-case associations of organized leisure-time activities (OLTAs) and risk of mental health problems (MHP) in 4,753 children. | | | | |
| --- | --- | --- | --- | --- |
|  | Model 1 OR (95%CI) | Model 2 OR (95%CI) | Model 3 OR (95%CI) | Model 4 OR (95%CI) |
| **Sport organized leisure-time activities** |  |  |  |  |
| Yes | **0.62 (0.51, 0.76)** | **0.62 (0.50, 0.77)** | **0.66 (0.53, 0.82)** | **0.64 (0.51, 0.80)** |
| No | ref | ref | ref | ref |
| *Nagelkerke R-Square* | 0.010 | 0.069 | 0.088 | 0.090 |
| **Non-sport organized leisure-time activities** |  |  |  |  |
| Yes | **0.72 (0.55, 0.93)** | 0.81 (0.62, 1.06) | 0.78 (0.59, 1.03) | **0.74 (0.56, 0.97)** |
| No | ref | ref | ref | Ref |
| *Nagelkerke R-Square* | 0.003 | 0.062 | 0.083 | 0.090 |
| Complete-case dataset consists of n=4,753 children. **Bold** indicates significance (p-value <0.05). Model 1 is a crude unadjusted model. Model 2 is adjusted for sociodemographic variables (i.e. age, gender (ref=boy), parental education (ref=higher), perceived financial difficulties (ref=no), family status (ref=two-parent), migrant status (ref=Dutch). Model 3 is model 2 and additionally adjusted for physical activity, and stressful life events (ref=no). Model 4 is model 3 and additionally (mutually) adjusted for sport or non-sport organized leisure-time activities (i.e. independent association). For this study we used data from a survey conducted between May-July in Rotterdam, the Netherlands from 5,010 children aged 4-12-years old. | | | | |

| **Table S3.** Complete-case associations of number of the breadth of organized leisure-time activities (OLTAs) and mental health problems (MHP) in 4,753 children. | | | |
| --- | --- | --- | --- |
|  | Model 1 OR (95%CI) | Model 2 OR (95%CI) | Model 3 OR (95%CI) |
| **Number of categories of** **organized leisure-time activities** |  |  |  |
| In 2-5 categories | **0.44 (0.30, 0.64)** | **0.50 (0.33, 0.74)** | **0.50 (0.34, 0.75)** |
| In 1 category | **0.60 (0.49, 0.74)** | **0.58 (0.47, 0.73)** | **0.62 (0.49, 0.78)** |
| None | ref | ref | ref |
| *Nagelkerke R-Square* | 0.015 | 0.072 | 0.091 |
| Complete-case dataset consists of n=4,753 children. **Bold** indicates significance (p-value <0.05). Model 1 is a crude unadjusted model. Model 2 is adjusted for sociodemographic variables (i.e. age, gender (ref=boy), parental education (ref=higher), perceived financial difficulties (ref=no), family status (ref=two-parent), migrant status (ref=Dutch). Model 3 is model 2 and additionally adjusted for physical activity, and stressful life events (ref=no). For this study we used data from a survey conducted between May-July in Rotterdam, the Netherlands from 5,010 children aged 4-12-years old. | | | |

| **Table S4.** Comparison between children with complete and missing data. | | | | |
| --- | --- | --- | --- | --- |
|  |  | Population for analysis (n=4,716) | Population excluded for analysis  (n=294) | P-value |
| **Gender** | Boy | 94% (2,428) | 6.0% (156) | 0.600 |
|  | Girl | 94% (2,288) | 5.7% (138) |  |
| **Family status** | Two-parent | 96% (3,581) | 3.7% (136) | **<0.001** |
|  | Single-parent or other | 90% (1,135) | 9.6% (121) |  |
| **Parental education** | Higher | 99% (2,429) | 1.2% (30) | **<0.001** |
|  | Intermediate | 98% (1,520) | 2.2% (34) |  |
|  | Lower | 96% (767) | 3.6% (29) |  |
| **Migrant status** | Dutch | 97% (2,219) | 2.9% (66) | **<0.001** |
|  | Non-Western migrant | 92% (1,886) | 7.8% (160) |  |
|  | Western migrant | 94% (611) | 6.4% (42) |  |
| **Perceived financial difficulties** | No | 94% (4004) | 4.6% (193) | <0.147 |
|  | Yes | 94% (712) | 5.8% (44) |  |
| **Physical activity, median (IQR)** |  | 1.7 (1.1-2.4) | 1.8 (1.1-3.0) | **<0.05** |
|  |  |  |  |  |
| **Current stressful life events** | No | 95% (3,564) | 5.3% (201) | 0.258 |
|  | Yes | 94% (1,152) | 6.2% (76) |  |
| **High risk of mental health problems** | No | 95% (4,289) | 4.9% (220) | **<0.001** |
|  | Yes | 90% (427) | 9.7% (46) |  |
| **Age, median (IQR)** |  | 8.0 (6.0-10.0) | 8.0 (6.0-10.0) | **<0.05** |
| **Sport organized leisure-time activities** | Yes | 96% (2,742) | 4.3% (123) | **<0.001** |
|  | No | 93% (1,974) | 6.8% (143) |  |
| **Non-sport organized leisure-time activities** | Yes | 94% (1,033) | 5.7% (63) | 0.495 |
|  | No | 95% (3,683) | 5.2% (203) |  |
| **Number of categories of** **organized leisure-time activities** |  |  |  | **<0.028** |
|  | In 2-5 categories | 96% (595) | 6.5% (25) |  |
|  | In 1 category | 95% (2,623) | 5.0% (137) |  |
|  | None | 94% (1,497) | 6.5% (104) |  |
| Row percentages are used. **Bold** indicates significance. Chi-square tests were used to test for differences in categorical variables. Valid percentages are reported. For this study we used data from a survey conducted between May-July in Rotterdam, the Netherlands from 5,010 children aged 4-12-years old. | | | | |
